# Patient Perspectives on a Targeted Text Messaging Campaign to Encourage Screening for Diabetes: Qualitative Study

**DOI:** 10.2196/41011

**Published:** 2023-01-17

**Authors:** Kristin M Lenoir, Joanne C Sandberg, David P Miller, Brian J Wells

**Affiliations:** 1 Department of Biostatistics and Data Science Public Health Sciences Wake Forest University School of Medicine Winston-Salem, NC United States; 2 Department of Public Health Sciences University of North Carolina at Charlotte Charlotte, NC United States; 3 Department of Family & Community Medicine Wake Forest University School of Medicine Winston-Salem, NC United States; 4 Department of Internal Medicine Wake Forest University School of Medicine Winston-Salem, NC United States

**Keywords:** mobile health, diabetes screening, electronic health records, text messaging, clinical decision support, mHealth, diabetes, mHealth intervention

## Abstract

**Background:**

A sizeable proportion of prediabetes and diabetes cases among adults in the United States remain undiagnosed. Patient-facing clinical decision support (CDS) tools that leverage electronic health records (EHRs) have the potential to increase diabetes screening. Given the widespread mobile phone ownership across diverse groups, text messages present a viable mode for delivering alerts directly to patients. The use of unsolicited text messages to offer hemoglobin A_1c_ (HbA_1c_) screening has not yet been studied. It is imperative to gauge perceptions of “cold texts” to ensure that information and language are optimized to promote engagement with text messages that affect follow-through with health behaviors.

**Objective:**

This study aims to gauge the perceptions of and receptiveness to text messages to inform content that would facilitate engagement with text messages intended to initiate a mobile health (mHealth) intervention for targeted screening. Messages were designed to invite those not already diagnosed with diabetes to make a decision to take part in HbA_1c_ screening and walk them through the steps required to perform the behavior based solely on an automated text exchange.

**Methods:**

In total, 6 focus groups were conducted at Wake Forest Baptist Health (WFBH) between September 2019 and February 2020. The participants were adult patients without diabetes who had completed an in-person visit at the Family and Community Medicine Clinic within the previous year. We displayed a series of text messages and asked the participants to react to the message content and suggest improvements. Content was deductively coded with respect to the Health Belief Model (HBM) and inductively coded to identify other emergent themes that could potentially impact engagement with text messages.

**Results:**

Participants (N=36) were generally receptive to the idea of receiving a text-based alert for HbA_1c_ screening. Plain language, personalization, and content, which highlighted perceived benefits over perceived susceptibility and perceived severity, were important to participants’ understanding of and receptiveness to messages. The patient-physician relationship emerged as a recurring theme in which patients either had a desire or held an assumption that their provider would be working behind the scenes throughout each step of the process. Participants needed further clarification to understand the steps involved in following through with HbA_1c_ screening and receiving results.

**Conclusions:**

Our findings suggest that patients may be receptive to text messages that alert them to a risk of having an elevated HbA_1c_ in direct-to-patient alerts that use cold texting. Using plain and positive language, integrating elements of personalization, and defining new processes clearly were identified by participants as modifiable content elements that could act as facilitators that would help overcome barriers to engagement with these messages. A patient’s relationship with their provider and the financial costs associated with texts and screening may affect receptiveness and engagement in this process.

## Introduction

### Background

The National Health and Nutrition Examination Survey showed that 14.7% of Americans aged 18 years and older met the criteria for diabetes, but an estimated 23.1% of those prevalent cases of diabetes are undiagnosed [1]. Although 38.0% of adults have prediabetes, only 19.0% reported being told that they had the condition by a provider [1]. Screening for high-risk patients using hemoglobin A_1c_ (HbA_1c_) is recommended by both the United States Preventive Services Task Force and the American Diabetes Association [2,3]. Detecting an elevated HbA_1c_ can provide an opportunity for intervention to prevent progression to diabetes and microvascular and macrovascular complications that accompany sustained elevated blood sugar levels [4,5]. Early detection is important because many patients with type 2 diabetes already have evidence of end-organ damage at the time of diagnosis [5-7]. Palladino et al [5] showed that 24% and 37% of incident cases already present microvascular and macrovascular complications, respectively, upon diagnosis.

Widespread adoption of electronic health records (EHRs) has created a framework in which a wealth of data can be synthesized to identify patterns and risks that may not be easily observable. We developed and validated a tool for using structured EHR data to identify patients at risk for an elevated HbA_1c_ [8] and intend to deploy this in clinical practice to flag patients without diabetes who might benefit from an HbA_1c_ test as part of a clinical decision support (CDS) process. CDS systems have become widespread across health care institutions and are intended to improve care delivery by integrating targeted information, often from underlying EHRs, to enhance medical decision-making processes [9]. In primary care, a CDS tool might provide an alert to a clinician to prompt an action that may benefit a patient’s health. This can be achieved through the delivery of education, medication optimization, or even ordering a screening test.

Although EHR-based CDS tools have shown small-to-modest improvements in clinical targets across a wide variety of settings and outcomes [10], they are subject to drawbacks [9,11]. Notable barriers to clinician-facing tools include alert fatigue [12], workflow disruptions, inconsequential alerts, and distrust [9]. Numerous studies report the great frequency with which clinicians override these alerts and ignore the requested action [13,14]. One way to overcome these barriers is to contact the patient directly and automate an action (eg, an HbA_1c_ test order) based upon their response, which can be achieved through a message via a health portal, phone call, or text message. Text messages present a cost-effective opportunity to reach all ages, races, and income groups, as 97% of Americans own a mobile phone [15].

### Text Messages

Within health care services, automated text messages have been widely used as reminders of medical compliance and upcoming appointments, which have yielded improved medical compliance and attendance [16]. Kitsious et al [17] found that mobile health (mHealth) interventions are better at improving glycemic control among those diagnosed with diabetes compared to non-mHealth approaches. Text-based mHealth interventions have increased medication adherence, self-management behaviors, and glycemic control among patients with diabetes [18]. In terms of mHealth’s role in screening practices, cancer screening has been positively impacted by texting intervention, although primarily through a reminder mechanism for decisions that had already been made [19]. Miller et al [20,21] showed that engaging patients overdue for colorectal screening with a patient-centered mHealth decision aid in a health care setting helped them decide to engage and follow through with colorectal screening in an automated fashion.

Studies examined in Haider’s systematic review [18] of effective texting interventions among patients with diabetes reflect messaging that occurred subsequent to both diabetes diagnosis and participants’ agreement to take part in the intervention. Selection bias may skew findings that patients are receptive to text interventions in diabetes management simply because they already knew of their health condition and demonstrated interest. What is unknown is how one might respond to an unsolicited, or “cold,” text that occurs outside of a health care context and whether it will be sufficient to encourage one to undergo HbA_1c_ screening. Cold texting, similar to cold calling, involves the generation of a text message to a patient who has not already agreed to receive messages as part of a study. HbA_1c_ screening for high-risk patients is considered a standard of care under the purview of broad consent for messages that patients agree to receive as part of their care. A literature review did not uncover similar cold-texting strategies and patient-directed texts to prompt HbA_1c_ screening.

### Conceptual Framework

The overarching goal of the text intervention was to facilitate patients’ decision and follow-through with a behavior of undergoing an HbA_1c_ test for the prevention and detection of diabetes and associated complications. We used the Health Belief Model (HBM) [22] to guide the development of text messages, as well as the content of a web link provided within text messages (Table 1). The HBM was originally established to explain why people engage in preventive health behaviors through the constructs of perceived susceptibility, perceived severity, perceived benefits, cues to action [22], and self-efficacy [23]. The HBM has since been widely used in preventive health behavior interventions [24,25] and is relevant to informing optimal text message content that facilitates receptiveness to and engagement with these messages.

**Table 1:** Development of text message intervention content and phrasing with respect to HBM^a^ constructs.

HBM construct	Text message phrasing/processes
Perceived susceptibility	We attempted to increase perceived susceptibility through using language such as “you may be at risk” and “your blood sugar levels may be high” and by indicating that they had not had a recent screening test.
Perceived severity	We limited text content related to perceived severity in consideration of the ethical implications of delivering alarming content without immediate access to a health care provider for discussion. We use phrases such as “this can lead to health problems” and included information about the importance of the detection and prevention of organ damage related to diabetes only in the web link content that had clear contact information for the research team, which included a physician.
Perceived benefits	We intended to increase perceived benefits through the use of language such as “take control of your health” and “you may benefit from” in text content and highlighted methods to manage health if the test was abnormal in the web link content.
Perceived barriers	We attempted to reduce the perceived barriers of cost, scheduling an appointment, and fasting before the test by indicating that the test is free (one can walk into a laboratory without scheduling an appointment/when it is convenient for them) and that there is no need to fast before the test in the text content.
Cues to action	The text message itself and follow-up reminders served as cues to action to trigger the decision-making process about going to a laboratory to get an HbA_1c_^b^ test.
Self-efficacy	We sought to increases one’s self-efficacy by providing concise but comprehensive and clear information about how participants could complete the steps required to engage in HbA_1c_ screening.

^a^HBM: Health Belief Model.

^b^HbA_1c_: hemoglobin A_1c_.

Key recommendations identified by an international workshop in the development of effective digital interventions that facilitate behavior change in health care include developing a person-centered approach in the design and development phase to inform how tools can be modified to meet user needs and preferences [26]. Soliciting feedback from stakeholders through iterative qualitative research is imperative to anticipate reactions and tailor content to promote engagement and accessibility [26]. Therefore, we sought feedback from a sample of patients who were eligible to receive these texts in the future. The purpose of this study was to gauge the perceptions of and receptiveness to text messages to inform the final crafting of content that would best facilitate engagement with text messages as the initial step in the decision-making process to take part in HbA_1c_ screening.

## Methods

### Recruitment

Potential focus group participants were identified through the institution’s EHRs as English-speaking adults (≥18 years old) with a lack of diabetes-related *International Classification of Diseases* (ICD) codes or diabetes-related medications, the presence of a mobile phone number in the EHR, and at least 1 in-person encounter within the Wake Forest Baptist Health (WFBH) Department of Family and Community Medicine within the past year. This particular clinic was used because clinicians agreed to implement HbA_1c_ screening using direct-to-patient alerts in the future. Strata were created by the unique combination of the following demographic categories: age (18-34, 35-49, 50-64, 65 years and older), sex (female, male), race (White, Black, other race), and ethnicity (non-Latinx, Latinx). Level of education and socioeconomic status (SES) were considered, but the lack of documentation in the EHRs precluded their use in stratification. Patients were selected from each stratum using a random number generator to increase the probability of participation among underrepresented groups. The intent was to contact equivalent numbers from each stratum. However, we were unable to do so because we exhausted lists of smaller strata before a sufficient number of individuals had committed to participate.

Randomly selected patients received a letter indicating that they may be eligible to participate, that they would be contacted by the study coordinator within 2-3 weeks to gauge interest, and that they could call the study coordinator directly to opt out or participate. The coordinator made 3 attempts to contact those who received letters by phone. For those who were interested in participating, eligibility criteria were verbally confirmed, which included never having been told that they had diabetes, owning a mobile phone, using text messages at least 5 times per week, and feeling comfortable sending and receiving text messages. Those who met the inclusion criteria were invited to participate in a focus group.

### Data Collection and Procedures

Data were collected from 6 focus groups formed between September 2019 and February 2020. The target number of participants for each focus group was 6-12. Of the 6 focus groups, 3 (50%) were held in the morning, 2 (33%) in the afternoon, and 1 (17%) in the evening in a familiar building where patients would typically attend appointments. Focus groups lasted 1.5-2.5 hours from sign-in to completion of all documentation.

The groups were led by a trained facilitator experienced in moderating group dynamics, who followed a semistructured focus group guide. A study member assisted the moderator with the Microsoft PowerPoint presentation of text messages and documented participants’ perceptions and observable body language. At the end of each session, we verbally summarized the range of expressed ideas and asked the participants to comment on validity [27].

The research team formulated initial potential text messages (Table 2) based on the HBM and preliminary input from researchers, clinicians, and patients. These messages were displayed for focus groups via a PowerPoint projection onto a large screen around which participants sat in a U shape to facilitate conversation. The participants were asked to tell us what they liked and did not like about the different messages and the process, including what they found confusing. The moderator solicited dissenting views and encouraged all attendees to participate. Suggestions for improvement were solicited, including perceptions of how their recommendations improved upon the text shown. Text messages were modified to incorporate this feedback for each subsequent focus group so that the original and modified texts were presented and discussed. All focus group sessions were audio-recorded and transcribed. Transcripts were compared with audio recordings for accuracy.

**Table 2:** Text message phrases presented to focus groups.

Type of message	Phrases^a^
Introductory phrases	Thanks for being a Wake Forest Baptist Health patient. An automated analysis of your Wake Forest health record indicates you may benefit from [a blood sugar screening test/glucose screening using Hemoglobin A_1c_]. Take Control of your health [today. Take a free test at Wake Forest Baptist Health/Take a free Hemoglobin A_1c_ test at Wake Forest Baptist Health/by getting a free blood sugar test]. An automated analysis of your Wake Forest health record indicates you may benefit from having a blood sugar screening test. WFBH^b^ has created a calculator that suggests your blood sugar levels may be high. A review of your medical records suggests you may be at risk of having a high blood sugar level. This can lead to health problems.
Additional information	Our records indicate that you have not had a [blood sugar screening/Hemoglobin A_1c_ blood test] in the past year. You can learn more [at/about a blood sugar screening test here/about A_1c_ testing at] (link). View (link) for more information.
Confirming interest or participation	Reply YES [to sign up/ to have the test/to have the test ordered/to schedule the test/if interested/to find out more/so we can order the test for you]. Reply NO to opt out.
Attending the laboratory	Great! The test has been ordered. Please stop by any Wake Forest Laboratory [at (link)] to have your blood drawn. You do NOT need to fast before the test. You can drop by any of our labs at your convenience for this free [blood sugar] test. Locations and hours [for labs/for Wake Forest labs] may be found at (link). You can drop by a lab at any time it is open.
Reminder message	Don’t forget to stop by [one of our labs/a Wake Forest Baptist Health Lab to have your (blood sugar screening test/A_1c_ test)]. Here is list of times and locations: (link). You can drop by a lab anytime it is open. Learn more at (link).
Results	Results will be sent to you using your current preferences [(MyWakeHealth or letter through the mail)]. Blood sugar levels outside the normal range will also be sent to your [primary care] physician.

^a^This summarizes the texts that were presented. Brackets indicate the different phrases used in conjunction with the overarching text message outside of those brackets, and forward slashes indicate the start and end of different phrases that were tested. For example, “Locations and hours [for labs/for Wake Forest Labs] may be found at (link).” This indicates that we tested both “locations and hours for labs may be found at (link)” and “locations and hours for Wake Forest Labs may be found at (link).”

^b^WFBH: Wake Forest Baptist Health.

### Analysis

We used deductive coding to distinguish transcript content associated with the HBM constructs. We used inductive coding to identify emergent themes that were discussed at length, which were also relevant to receptiveness to and engagement with text messages. We created a codebook with themes and definitions after an initial review of focus group transcripts and refined definitions during the coding process [28]. Two team members independently applied the codes or tags to segments of the transcripts related to emergent themes [29] using ATLAS.ti v.7 software (Scientific Software Development GMBH, Berlin). We continued to implement focus groups until thematic saturation was reached. We extracted segments of text from the transcripts and organized these excerpts with respect to text messages and themes to facilitate the identification of concepts across focus groups. Quotations in this manuscript are labeled with the focus group number (FG1-FG6, sex [M: man, W: woman], Mod: moderator) and participant identification number within each relevant focus group.

### Ethical Considerations

This study was approved by the Institutional Review Board of Wake Forest University Health Sciences (IRB00041549) and was conducted in accordance with the Declaration of Helsinki and all other relevant guidelines and regulations. Written informed consent was obtained in person from each participant in a 1-on-1 setting before the start of the focus group. Participants in the first 4 groups received a US $25 gift card for their participation. Considering modest recruitment and high no-show numbers, a US $50 gift card was offered for the last 2 focus group sessions. Participants completed an anonymous paper-based survey upon completion of the focus group sessions to ascertain demographic characteristics and texting habits. Transcripts of focus group sessions were de-identified.

## Results

### Focus Group Characteristics

A total of 405 recruitment letters were distributed among the eligible population of 3580 individuals who were identified using EHRs. Of these, 65 (16%) signed up for a focus group session and 32 (7.9%) followed through with participation. Four additional participants who met the inclusion criteria were recruited through word of mouth from other potential participants and were permitted to attend a session because those who had previously committed dropped out at the point of the coordinator’s confirmation call.

In total, 36 participants attended 1 of 6 focus group sessions. Almost half of the participants (n=16, 44%) identified as a race other than non-Latinx White, and 20 (56%) of participants were female (Table 3). All age groups were represented, and most were aged 50 years or older. The sample was highly educated, with 24 (67%) having a bachelor’s degree or higher. All participants engaged in regular daily texting habits: half (n=18, 50%) sent 2-9 texts per day, and most (n=17, 47%) received 10-50 texts per day.

**Table 3:** Characteristics of focus group participants (N=36).

Characteristics		Participants, n (%)^a^
**Sex**		
	Female	20 (56)
	Male	16 (44)
**Race/ethnicity**		
	White/Caucasian, non-Latinx	20 (56)
	Black/African American, non-Latinx	15 (42)
	Other, non-Latinx	1 (3)
**Age (years)**		
	18-34	10 (28)
	35-49	4 (11)
	50-64	12 (33)
	65 or older	10 (28)
**Education**		
	High school	2 (6)
	Associate’s degree or some college (no degree)	10 (28)
	Bachelor’s degree or higher	24 (67)
**Texts sent per day**		
	0-1	0
	2-9	18 (50)
	10-50	17 (47)
	>50	1 (3)
**Texts received per day**		
	0-1	1 (3)
	2-9	15 (42)
	10-50	19 (53)
	>50	1 (3)

^a^The percentages might add up to more than 100 because of rounding.

### HBM-Related Discussion

#### Perceived Susceptibility, Severity, and Benefits

Messages that contained the word “risk” were largely off-putting for participants across focus groups. For example, 1 participant noted that they “…don’t like when they’re trying to scare you into something” (FG5F1), and many others agreed that language that indicated that “risk of high blood sugar that could lead to health problems” would be “frightening.” A couple of participants indicated that they would want to immediately call the medical office, speak to someone, or “schedule an appointment…to figure out what [their] blood sugar situation is” (FG6M1).

So the first thing I am going to do myself is contact my family physician about this text I just received right here. Do you feel I should come in and have a blood sugar test because I got a text saying I could be at high risk, but you haven't checked my blood sugar?

FG2M2

In contrast, some participants liked the idea of describing risk and cited that it would give people “motivation” to act or that it would make them concerned as opposed to scared. Two participants in one focus group indicated that they wanted to know the dangers associated with high blood sugar levels and were supportive of including additional information in the web link.

The majority of participants stated that they preferred the texts containing language that highlighted perceived benefits, such as “take control of your health” or “you may benefit from,” as opposed to language that elevated perceived susceptibility and severity, such as “…something that's not scary and that is like, that is kind of positive in terms of how it's presented, but not in a way that is like you're a child” (FG3M3). One person suggested:

If you put a recent analysis of your Wake Forest health records indicating you might be benefit from blood sugar screening as a form of preventive medicine, or preventive test, something like that, I can’t think of the word right now, but in that aspect they might make somebody more comfortable looking at it’s a preventive procedure, and not that “I’ve got something right now.”

FG1M2

This statement was affirmed verbally and through head-nodding gestures by additional participants.

#### Perceived Barriers

Participants discussed that some of the texts appeared “spammy,” but they did not discuss the privacy of their medical information as an issue. Fiscal costs were discussed, and participants across focus groups affirmed the importance of conveying the test was free and suggested that we reiterate “free” in the follow-up text messages confirming the order and reminders to stop by the laboratory. The cost of text messaging was perceived as a barrier to engaging with text messages for some. One participant said that their limited phone plan would cause them to ignore the text messages. One person was happy that they did not need to fast for the test. The ability to walk into any laboratory at their convenience was also favorably received, although a minority wanted to make an appointment for the test or desired specific instructions on how and when to proceed because they were unsure of what would happen. One person indicated that the automated nature of the process would be a barrier:

I wouldn't be okay with it only because I haven't talked to someone. It's all been automated.

FG1F2

#### Cues to Action and Self-efficacy

Participants discussed receptiveness to this text messaging strategy and indicated that their engagement with texts and follow-through with testing may be contingent on the information presented, which is further elaborated upon in the Emergent Themes section.

Mod: So just in general, how would you feel about getting a set of messages somewhat like these that have been made a little bit better to address your comments and concerns? Does it seem like a good thing, does it seem like what in the world is going on?

FG3F1: I'm good.

FG3M1: I think if they were worded correctly, I think I would appreciate it.

FG3F2: Same.

FG3M2: Me too.

FG3F2: Yeah, it's good preventative health.

All focus groups discussed that they approved of follow-up texts if there were no responses, and reminders that their test was ready. Additionally, multiple participants cited illness or diabetes in a family member or their role as a caregiver as a reason why they would engage with text messages as a cue to take action to undergo an HbA_1c_ test. Overall, many patients appeared to be receptive to the texting process and had additional thoughts that could affect the receptiveness and engagement with texts, as discussed in the Emergent Themes section of this paper.

### Emergent Themes

#### Language

Language was discussed as a potential barrier and facilitator of engagement and understanding. Across focus groups, participants overwhelmingly indicated that there were words that they did not understand in a subset of text messages. There was a preference for plain language, such as “blood sugar test” over “hemoglobin A_1c_ test.” One person indicated that “[A_1c_ test] doesn’t mean anything to me” (FG6M2). Another indicated that “most people do not know what [an A_1c_ test] is” (FG6F2). Although a blood sugar test is slightly different from an HbA_1c_ test, many participants knew that it is related to diabetes. The words “automated,” “computerized calculator,” and “analysis” in the context of describing how patients were selected to receive messages elicited overwhelmingly negative responses across all focus groups as it was “not personal,” “unnecessary,” or “big brother-ish.”

FG1M3: With all them robo-calls going on, I really don't want to see automated.

[Laughter]

FG1F1: The robots are watching. Which is kind of what is, but…

Mod: Others—what do you think of that? Does automated analysis make you think “Oh, that's great - it means this is really sophisticated” or does it send chills up your spine?

FG1F1: No, not for me. It makes me paranoid…Why is the robot reading my records?

FG1F2: Someone else is looking at my records? Who is reading my records besides my doctor?

The word “calculator” was cited as “strange,” “doesn’t fit,” and “confusing.” One person described that the word “automated” as seeming like “…you threw a bunch of names in a bucket.” Another indicated that it would cause them to think the selection was “random,” and another described it as “a telemarketer type system.” The majority of participants across all 6 focus groups were more receptive to language similar to “a review of your record/chart” or “your health record indicates” that you may benefit from a blood glucose test. A review of the record added a bit of “urgency” for 1 person in contrast to thinking about an “algorithm in the background.” Multiple participants indicated that this made them feel that someone was looking at their chart to select them, which was more favorable.

#### Personalization

Personalization of text message content emerged as an important condition for deciding to engage with text messages and accessing the web link embedded in the text. Many participants questioned whether these texts would actually pertain to them. Some participants wanted their name, the department name, or an indication that their doctor was involved in order to pay attention to the text as opposed to a header of “Wake Forest Baptist Health,” which might cause them to ignore the text because it doesn’t “have anything to do with [them]” and it’s “random” or “coming from a complete stranger.”

FG4F1: I mean, it says Wake Forest Baptist at the top, but if it specified my doctor's office, I might actually take it more seriously. Yeah, but I feel like that's more of a generic like, yeah, this is a scam.

Mod: So are you thinking your doctor's name or your Family and Community Medicine be adequate?

FG4F1: Probably Family and Community Medicine. Like I don't actually come to Family and Community Medicine, I'm at Peace Haven Family Medicine. So if it came from Peace Haven Family Medicine.

Mod: Okay. How about if it said from your family medicine, practice at Wake Forest or family medicine doctor, would that help at all or would you still feel like that's too generic?

FG4F1: Yeah, I don't know.

FG4M1: I think it should come from the doctor, you know, maybe have the doctors name possibly. Because you get a lot of text messages just in general.

There were mixed opinions, as others said that a simple header of “Wake Forest Baptist Health” or the text coming from the “known phone number” used by Wake Forest Baptist Health would be sufficient for them to pay attention to the message because they already receive appointment reminders and phone calls from that number.

One person indicated that they would be more likely to respond if the message comes through the MyWakeHealth portal because “they check that” and it “makes it feel legit” since they already get messages indicating they need things, such as a flu shot. Although privacy and security were not as much of a topic of discussion as we anticipated, participants noted that elements of personalization would help them overcome the fear that this might be a “scam” or “spam” and may make them more likely to click the link to additional information embedded in the text.

#### Patient-Provider Relationships

Numerous participants made assumptions about their provider’s participation in the texting and testing process. Participants across 4 focus groups assumed that their primary care physician (PCP) would be involved in the initial delivery of text messages by reviewing their records or requesting that they receive the text message. One person noted that they thought the phrase “might benefit from [a screening test]” automatically meant that their “...provider thinks that I should have this done” (FG6F2). Another participant indicated that they wanted to “…feel like maybe [their provider] was involved [in the process of selecting them]” (FG5M1). Respondents across multiple focus groups indicated that they would wonder why the text message would be the first time they would hear about the issue instead of during a doctor’s visit. One person noted that this process might not work for someone who does not have a close relationship with their doctor. Multiple participants said that they would like to see their doctor’s name or a specific practice name in the text content instead of the overarching “Wake Forest Baptist Health.”

#### Mechanics of Undergoing the HbA_1c_ Test

Lack of clarity was discussed as an issue that could lead to frustration with the text messages and the process. After a patient replied in favor of an HbA_1c_ test, they would be able to walk into any WFBH-affiliated laboratory during regular hours, check in, and promptly undergo a blood draw that is necessary for the test. Respondents across all focus groups indicated that this was unclear. Upon replying “yes” to have the test ordered, many participants assumed that there would be an appointment time associated with a lab visit. Multiple people assumed that someone would call them to schedule the laboratory test, and a few indicated that they might be reluctant to participate because they inferred an automatic assignment to an appointment time without consulting whether it would work with their schedule. Another person wondered whether a laboratory site would be assigned to them. Another assumed that setting up the appointment time would require an additional set of text messages and expressed potential frustration with numerous text transactions. A few participants preferred a scheduled appointment as opposed to a walk-in because they would be more likely to put it on their “regimented” schedule and would know the laboratory staff would expect them.

If I don't have a set commitment in timeframe, it's not going to happen. It's just not. So that, in the giving the option for an appointment might be better I realized that this is about people being able to just walk into any lab, but there's also the matter of just some people aren't going to be able to work that out unless there is a set schedule. I have to have everything regimented personally. So it's like, that's just me though.

FG3M3

And me too. And, maybe if people don't respond or don't show up and get the test, then maybe a second set of text could be sent out to them. I don't know how complicated it is to have it be like take you to setting up an appointment or, but I mean, yeah, I think you, you're probably, you might lose some people who don't get around to things whoever, yeah.

FG3F1

I think also that it's common for people if they schedule a time to, exercise or whatever, they're more apt to do it than to just say, you, I'll do it sometime today or sometime this week, or go extra, go for a walk. Or I think that is kind of human nature if it's Wednesdays at 4:30 I go do this, then you show up for.

FG4F1

Many participants assumed that they would need to go to the lab at their PCP’s office and were unaware that the health system had multiple labs. Once they were informed about their ability to attend any lab at their convenience, some participants expressed confusion about what they should do upon arrival. Would the staff understand why they were there, or would they have a particularly long wait because they did not have an appointment time?

Well in most people's experience if they show up just walk into any kind of lab or doctor location and they're not going to get anything.

FG6M3

Once we explained the process and that a link to laboratory information (location, hours, and phone number) was provided in the supplemental website, participants were receptive, and 1 indicated, “That’s brilliant! You need to get that across better though” (FG5F3).

#### Receiving Results

Three focus groups discussed lack of clarity about how they would receive results once they had their test. The WFBH delivers results of normal lab tests by the patients’ preferred mechanism that is documented in the EHRs (MyWakeHealth portal, phone, or mail), and providers may reach out by multiple mechanisms if it appears that the patient cannot be reached. The phrase “current preferences” was insufficient to convey this. Many participants assumed that they would get a follow-up call if their results were abnormal or knew that this was standard a practice at WFBH, and a few thought they would get a call from their provider. Some participants assumed that the results would go in their medical records and that only abnormal results would be flagged, and they suggested language indicating that results would be sent to their physician, who would follow up. Another was worried that they might not get the results because they had issues in the past when medical material was sent to an old address.

## Discussion

### Principal Findings

We sought to use patient feedback to optimize content that promotes receptiveness to and engagement with text messages intended to initiate decision-making to engage in HbA_1c_ screening. Positive language that highlighted perceived benefits was preferable to language emphasizing perceived risk and severity in most focus groups. Participants frequently acknowledged that the perceived barrier of test costs was reduced by repeatedly indicating that the test was free. Plain language, a key component of patient accessibility [30,31], and personalization, a known facilitator of behavioral intention [32], emerged as themes identified by patients that could overcome barriers to engagement with text messages. Confusion about attending the laboratory to undergo the HbA_1c_ test and receiving results were the most cited themes that required further clarification in text content. Finally, patient-physician relationships appeared to be an important and recurring influence in multiple aspects of the process and may affect whether one would be responsive to text messages and this method of soliciting patient engagement.

### Tension Between “Precision Medicine” and “Personal Medicine”

Advanced data analytics now allows health care providers to tailor treatments to individual patients, an approach referred to as “precision medicine” [33]. Similarly, health care systems can use analytics to identify populations of patients most likely to benefit from screening. We assumed that patients would value this precision medicine approach. However, we found that participants were far more receptive to content when they perceived some sort of connection with their provider and had negative responses to messages that lacked this connection, which reflects other mHealth research [34]. This seems to reflect a tension that exists between patients’ awareness of the potential benefits of an automated process that identifies those who might benefit from screening and their desire to receive health care through a provider they know and trust.

Participants mentioned the role of their own provider in a number of contexts, including how they would be selected, how they would get the test, and what would be done after the test. It was apparent that many of the participants placed a great deal of trust in their provider and that language that made them feel like the provider was more involved was desirable and elevated the perceived benefits of HbA_1c_ screening because it made them feel like “my doctor thinks I should [get the test].” Patients’ trust in their provider is positively associated with protective health behaviors, better quality of life, and satisfaction with care [35], and it is possible that the relationship built from face-to-face interactions may extend to or modify automated mHealth interactions with health care organizations in general. This is similar to findings indicating that trust in the health care system is 1 of the most important aspects of patient acceptance of and perceived benefits from artificial intelligence [36]. We might anticipate that those with established and positive relationships with their providers may engage more with text messages and other automated methods.

### Confusion and Uncertainty Surrounding Novel Methods

Participants were noticeably accustomed to events that occurred in a certain way and order. An anticipated routine of preventive care is (1) the patient attends an appointment; (2) the provider discusses important health issues at that appointment; (3) the provider orders tests, for which either the patient conveniently goes down the hall to complete immediately or the patient schedules an appointment to complete additional testing; and (4) the provider follows up with the results of the tests via a message or a phone call. Breaking that routine created uncertainty in each analogous step of this process in the text messaging scenario, which indicated that we must be deliberate with phrasing to convey sufficient information to decrease frustration with text messages and increase self-efficacy in undergoing HbA_1c_ screening sans an appointment and provider interaction. Unclear or insufficient information could adversely affect receptiveness to and engagement with text messages. Although text messaging limits the amount of content that can be provided, a link to a web page can detail why patients received the text and additional information needed to successfully follow through, such as a list of laboratory locations.

The adoption of new technology and processes is often accompanied by barriers, including technical challenges and resistance to change, as evidenced by the introduction of telehealth [37] and contact tracing [38]. Although texting has become normalized and effective for diabetes management [18] and for reminding patients about appointments [16], the novelty of this cold-texting process is that patients do not have prior knowledge of this alert and activities in which they are being asked to engage. The hope is that this process will become common for similar health-related activities that are considered standard of care and that the themes identified in this paper can inform future tools to optimize receptiveness to and engagement with text messages in different fields. These results are also applicable to patient-centered care, in which patients are given an opportunity to engage in clinical decisions that affect their health but need appealing and adequate information to do so, especially in direct-to-patient alerts that bypass in-person office visits.

### Limitations

One limitation of this study is selection bias, although we chose participants from a population that would be eligible to receive text messages in the future and attempted to sample equally across demographics. Those who elected to participate in the focus group may have had resources and time to attend or interest in mHealth or diabetes and therefore might not fully characterize the eligible population at WFBH. In particular, those with a high school education or lower and those who identified as Latinx were not well represented, which may have affected the presence of emergent themes. For example, although we strived to maintain simple language, those with lower SES or education may have had additional difficulty with text message content that was not identified in this study. Additionally, data collected during face-to-face, focus group discussions is subject to self-report and social desirability bias. The moderator actively elicited different opinions by asking for different perspectives and calling on specific individuals to attenuate group effects, such as when one participant voices an idea and others may be more likely to affirm the position. Finally, the data were collected from a relatively small number of participants in 1 geographic region and therefore may not be generalizable to other regions.

### Future Directions

One reason for proposing this texting approach is physician overload and unresponsiveness to EHR-based alerts. In addition to removing physician burdens, automated systems could help ensure that patients are receiving appropriate guideline-driven therapies and alert them to items their provider may be unable to identify. The next step in the process is to use optimized text messages and web content in accordance with what we discovered in these focus groups by emphasizing the benefits of screening, using plain language, adding elements of personalization, and clarifying processes. Ensuring optimal text message content is the first step in the overarching goal of having the patient follow through with glycated hemoglobin screening. Future research is necessary to determine whether there will be adequate engagement with these messages, whether this process will lead to meaningful health behaviors (HbA_1c_ screening), and whether this could apply to other health-related opportunities.

### Conclusion

Our findings suggest that patients may be receptive to text messages that alert them to a risk of having an elevated HbA_1c_ in direct-to-patient alerts that use a cold-texting approach. Using plain and positive language, integrating elements of personalization, and defining new processes clearly were identified by participants as modifiable content elements that could act as facilitators to help overcome barriers to engagement with these messages. A patient’s relationship with their provider and the financial costs associated with texts and screening may affect the receptiveness and engagement in this process.

## Abbreviations

CDS: clinical decision support
EHR: electronic health record
HbA_1c_: hemoglobin A_1c_
HBM: Health Belief Model
mHealth: mobile health
PCP: primary care physician
SES: socioeconomic status
WFBH: Wake Forest Baptist Health

## Data Availability

The data sets generated and analyzed during this study are not publicly available due to the discussion of potentially sensitive health-related information in transcripts. Data are available from the corresponding author on reasonable request and with appropriate approvals.
